# AWE-INDUCTION IMPROVES RECOVERY FROM STRESS AND IMPROVES COGNITIVE PERFORMANCE

**DOI:** 10.1093/geroni/igae098.3636

**Published:** 2024-12-31

**Authors:** Kristina Hash, S Alison Bolling, Leslie Tower, Julie Hicks Patrick

**Affiliations:** West Virginia University, Morgantown, West Virginia, United States; WVU, Morgantown, West Virginia, United States; WVU, Morgantown, West Virginia, United States; West Virginia University, Morgantown, West Virginia, United States

## Abstract

Portable, affordable and nonpharmacological interventions are needed to support physical and cognitive wellbeing across the lifespan. A promising psychosocial intervention to accomplish such goals includes awe inductions. Using our previously-validated video inductions, data are provided by 61 adults (M age = 28, range 18-81 yrs). Continuous measures of heart rate variability (HRV), including respiration, Baevsky’s stress index and other indices of sympathetic and parasympathetic functioning were collected via Polar H10 heart rate monitors. Participants completed flanker and choice reaction time (RT) tasks. Manipulation checks verified adherence to the protocol. As expected, performance was slower in the cognitively challenging incongruent flanker conditions relative to the easier congruent condition. A series of moderated regressions examined whether age altered the relations between HRV and cognitive performance. Differences in Baevsky’s stress index between the Neutral and Awe conditions interacted with age to explain 27% of the variance in post-awe incongruent flanker reaction time, F (3, 51)= 6.30, p<.001. Both age and the interaction term uniquely contributed to the equation. Probing the interaction revealed that although younger adults increased their speed of response under the awe condition, older adults increased their accuracy and slowed their response times accordingly. HRV indices suggested that across age, the awe induction resulted in decreased parasympathetic functioning, suggesting that awe might improve health and recovery from stress.

